# Accounting for soil moisture improves prediction of flowering time in chickpea and wheat

**DOI:** 10.1038/s41598-019-43848-6

**Published:** 2019-05-17

**Authors:** Yashvir S. Chauhan, Merrill Ryan, Subhash Chandra, Victor O. Sadras

**Affiliations:** 1 Department of Agriculture and Fisheries (DAF), Kingaroy, Queensland 4610 Australia; 2 DAF, Hermitage Research Station, 604 Yangan Road, Warwick, Queensland 4370 Australia; 3 Agriculture Research Division, Agriculture Victoria, 255 Ferguson Road, Tatura, 3616 Victoria Australia; 4 South Australian Research and Development Institute and School of Agriculture, Food and Wine, The University of Adealide, Adelaide, Australia

**Keywords:** Plant physiology, Abiotic

## Abstract

Matching crop phenology to environment is essential to improve yield and reduce risk of losses due to extreme temperatures, hence the importance of accurate prediction of flowering time. Empirical evidence suggests that soil water can influence flowering time in chickpea and wheat, but simulation models rarely account for this effect. Adjusting daily thermal time accumulation with fractional available soil water in the 0–60 cm soil layer improved the prediction of flowering time for both chickpea and wheat in comparison to the model simulating flowering time with only temperature and photoperiod. The number of post-flowering frost events accounted for 24% of the variation in observed chickpea yield using a temperature-photoperiod model, and 66% of the variation in yield with a model accounting for top-soil water content. Integrating the effect of soil water content in crop simulation models could improve prediction of flowering time and abiotic stress risk assessment.

## Introduction

The world faces the growing challenge of feeding over 9.5 billion people by 2050 under the looming threat of climate change^1^. To address this challenge while reducing the carbon footprint and conserving water, our reliance on protein from plants will need to increase significantly^2^. Chickpea is the third most important protein rich grain legume which is directly consumed as human food in the poorer countries where the projected population increases are most likely to occur^3^. The crop is also important for sustainability of farming systems due to its nitrogen fixing ability^4^. Chickpea yields are constrained by the crop’s high sensitivity to a number of abiotic stresses including frost, drought and heat stress^5,6^.

Matching crop phenology to environment is critical for stress adaptation and crop yield^7^, hence the importance of accurate prediction of flowering time. Based on experimental studies^8,9^, crop simulation models including the Agriculture Production Systems Simulator (APSIM) and Decision Support System for Agrotechnology Transfer (DSSAT)^10–13^, model chickpea phenology as a function of temperature and photoperiod. However, prediction of flowering time based on these two parameters is relatively poor^14–16^, suggesting other drivers of crop development may have been overlooked.

A few studies have shown that soil water can influence flowering time in chickpea and wheat. Kwang-Wook^17^ and Singh^18^ reported a positive relationship between chickpea flowering time and crop evapotranspiration. Singh^18^ reported that the thermal time requirement for emergence to flowering and flowering to maturity of chickpea decreased as normalized evapotranspiration deficit increased. Johansen, *et al*.^19^ also reported differences in flowering time between irrigated and rainfed chickpeas. A similar effect of soil water deficit on flowering time has been reported for wheat^20–22^. In wheat, photoperiod and vernalisation genes only accounted for about 53% of the variation in flowering time^23^, indicating that a significant proportion of the observed variation could be due to other factor(s), including soil water.

Limited attempts have been made to incorporate the soil water effect in models for flowering time, with emphasis on the drier end of the soil water range^21,22,24^. The APSIM model has an option to delay flowering of maize, sorghum and peanut in dry soil (www.apsim.info). Here we propose that the soil water effect on flowering time in chickpea and wheat can involve both (i) a hastening effect of water deficit, and (ii) a delaying effect of wet soil. Both interpretations have been advanced in the literature, but the former dominates^18,19^. The possibility of soil water delaying flowering in wheat has been alluded to by McDonald, *et al*.^25^, but this was not investigated further. This study describes the detection of the dynamic effect of high soil water on flowering time and its use in the APSIM model to improve flowering time prediction. Our focus is chickpea, with a secondary analysis in wheat.

## Results and Discussion

### Detection of soil water effect on flowering time of chickpea

**Figure 1:**
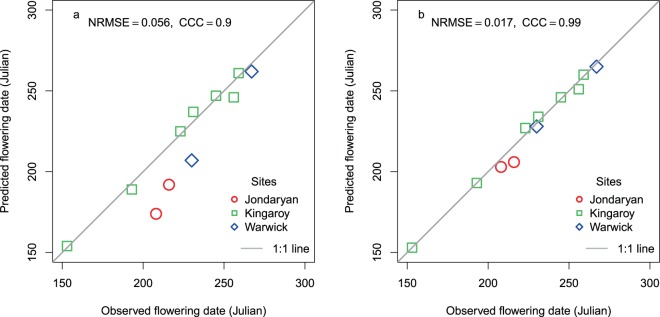
Observed and predicted flowering times (**a**) using the temperature (t) and photoperiod (p) based model, and (**b**) improved model based on t, p and soil water. NRMSE is the normalised root mean square error. The grey line is y = x. CCC = Lin’s concordance correlation coefficient.

Prediction of flowering time using temperature and photoperiod^10^ for 11 sowings at three sites in northern Australia resulted in a discrepancy of up to 34 days between the observed and predicted flowering times (Fig. 1a). The magnitude of this discrepancy was about 1.5 times larger than the range of genetic variation in flowering time in diverse field environments across Australia^26^; hence the importance of understanding the source of this discrepancy. Previous studies in chickpea have suggested that soil water deficit can advance flowering time^17,18^. Since the three soils in our study varied in plant available water holding capacity, we suspected that soil water could have caused this discrepancy. The maximum discrepancy occurred at Jondaryan and Warwick in South East Queensland, Australia where chickpea crops were grown with considerable sowing to pre-flowering rainfall (Table 1) on Vertisols with higher water holding capacity.

**Table 1:** Location, date of sowing, soil type, plant available water holding capacity (PAWC), row spacing, starting profile water, pre-flowering rain and irrigation in 11 sowings.

Location	Sowing date	Soil (APSoil no)	PAWC	Row spacing	Starting water^a^	Pre -flowering rain^b^	Irrigation
			**mm**	**cm**	**%**	**mm**	**mm**
Kingaroy	22-May-14	Ferrosol (107)	109	50	12	38	120
Kingaroy	12-Jun-14	Ferrosol (107)	109	50	12	77	80
Kingaroy	3-Jul-14	Ferrosol (107)	109	50	12	88	40
Kingaroy	23-Apr-15	Ferrosol (107)	109	90	7	71	50
Jondaryan	24-Apr-15	Vertisol (523-Generic)	136	90	7	115	0
Warwick	27-Apr-15	Brown Vertosol (33)	109	76	12	100	0
Kingaroy	4-Apr-16	Ferrosol (107)	109	90	32	9	30
Jondaryan	17-Apr-16	Vertisol (622-YP)	257	90	23	83	0
Kingaroy	12-Jun-17	Ferrosol (107)	109	90	23	77	25
Jondaryan	17-Jun-17	Vertisol (30)	244	90	17	54	60
Warwick	26-Jun-17	Brown Vertosol (33)	109	76	32	22	0

^a^Starting water was initialised in the model on 1^st^ Nov of the previous year. ^b^Sowing to pre-flowering rainfall. APSoil data are available from the google map at http://apsrunet.apsim.info/.

To understand how soil water status can influence flowering time of chickpea we modelled its effect. This was challenging because soil water availability is highly dynamic, as it is influenced by crop (i.e. size and functionality of canopy and root system), management (e.g. row spacing), soil properties and weather. Our data set representing a range of water holding capacities (109 to 257 mm) associated with site-specific soils (Ferrosol, several Vertisols), row spacing and rainfall (Table 1), captured many of these effects. In the absence of soil water measurements, each of the 11 simulations commenced with a low starting available water on 1^st^ Nov of the previous year, which was about six months before sowing. This starting water was determined using the Australian Landscape Water Balance model accessed at www.bom.gov.au/water/landscape. We addressed the challenge by developing a simple equation (Eq. 1 described in methods) that captured the dynamic relationship between fractional available soil water (FASW), which is the ratio of available water to the total available water, and daily thermal time (TT). The equation was developed through manual adjustments in daily thermal time when simulated FASW was >0.65 to fit the observed flowering data. An FASW of >0.65 represents the readily available water in the surface 0–60 cm layer where most roots are present^27^. This equation improved the Lin’s Concordance Correlation Coefficient (Lin’s CCC), which measures a model’s predictive performance^28^ as defined in Eq. 6 in Methods, to the almost perfect (>0.99) category (Fig. 1b). Applying this relationship below or above 60 cm increased the normalised root mean square error (NRMSE).

**Figure 2:**
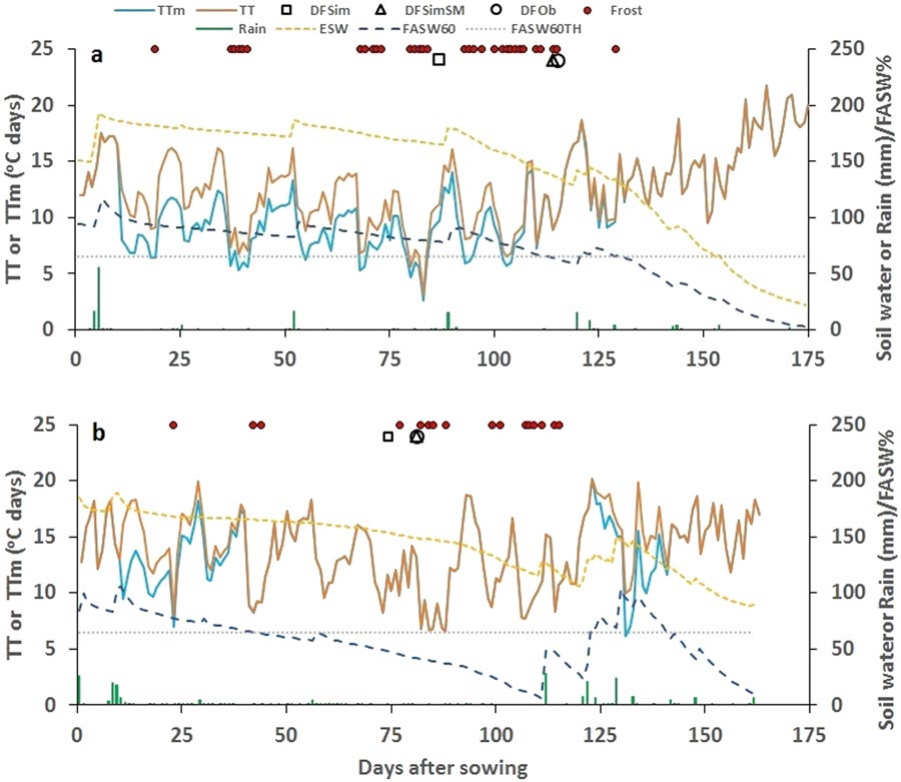
Dynamics of extractable soil water (ESW), rain, daily thermal time (TT), modified thermal time (TTm), flowering time predicted with soil water (DFSimSM), and without soil water input (DFSim), observed days to flowering (DFO), and frost events at Warwick (**a**) and Kingaroy (**b**) in Queensland in 2015. Kingaroy also received 25 mm irrigation (shown as rain) given at sowing and 113 days after sowing. FASW60 is fractional available soil water (FASW) of 60 cm layer (%) and FASW60TH = FASW threshold (%).

The relationship between soil water and thermal time espoused through Eq. 1 modifies thermal time accumulation to influence flowering time in different environments depending upon their soil and climatic attributes. In the 11 sowings, we observed a much larger delay in flowering time in self-cracking clay Vertisols of Warwick and Jondaryan in which the plant available water holding capacity is higher as compared to Ferrosols of Kingaroy. Comparison of soil water changes in 2015 at Kingaroy and Warwick suggested, that over time, FASW declined more gradually at Warwick as compared to Kingaroy (Fig. 2). The modification of thermal time at Warwick was therefore greater and occurred over a longer period resulting in slower accumulation of thermal time target of about 1017 °Cd for flowering compared to 1048 °Cd for Kingaroy. The difference in achieving these targets was consistent with about a month delay in flowering observed at Warwick.

**Figure 3:**
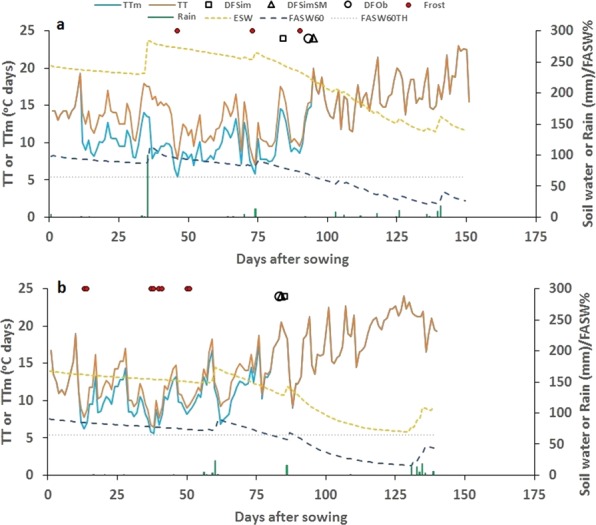
Dynamics of extractable soil water (ESW), rain, daily thermal time (TT), modified thermal time (TTm), flowering time predicted with soil water (DFSimSM) and without soil water input (DFSim), observed days to flowering (DFO), and frost events in 1979 (**a**) and 1980 (**b**) chickpea crops of cultivar Tyson near Dalby, Queensland (data from Beech and Leach)^29^. FASW60 is fractional available soil water (FASW) of 60 cm layer (%) and FASW60TH = FASW threshold (%).

To explore the effect of pre- and in-season rainfall on the same soil, we re-analysed the data of Beech and Leach^29^ for chickpea cultivar Tyson, sown in 1979 and 1980 at Dalby, Queensland in which soil water measurements were also made (Fig. 3). The 1979 season was wetter than 1980 resulting in higher soil water in the surface layers^30^. In 1979, total water use was 191 mm by 28^th^ Aug and 279 mm by 26^th^ Sep. In 1980, total water use was 93 mm by 1^st^ Sep and 161 mm by 30^th^ Sep. The soil water use simulated in 1979 was 179 mm and 268 mm on 28^th^ Aug and 26^th^ Sep, respectively, and in 1980, 106 mm and 149 mm on 1 Sep and 30^th^ Sep, respectively. The crops flowered 94 days after sowing in 1979 and 83 days after sowing in 1980. Days to flowering simulated without the input of soil water was 83 in 1979 and 84 in 1980. With the soil water input, days to flowering time simulated was 94 to 96 in 1979 and 81 to 84 in 1980 for a range of row spacing and planting densities used.

A larger modification of daily thermal time due to higher extractable soil water and over a slightly longer period was evident in 1979 (Fig. 3), which could have contributed to the delayed achievement of thermal time target for flowering as compared to 1980. The cumulative (unadjusted) thermal time targets computed by adding soil water effect for 1979 and 1980 were 1244 and 1035 °Cd, respectively, which closely matched the observed 1214 and 1032 °Cd reported in the study^29^. The increase in thermal time target for flowering with higher soil water was consistent with similar differences observed in irrigated and rainfed chickpea on a Vertisol soil in India^31^.

### Agronomic implications

**Figure 4:**
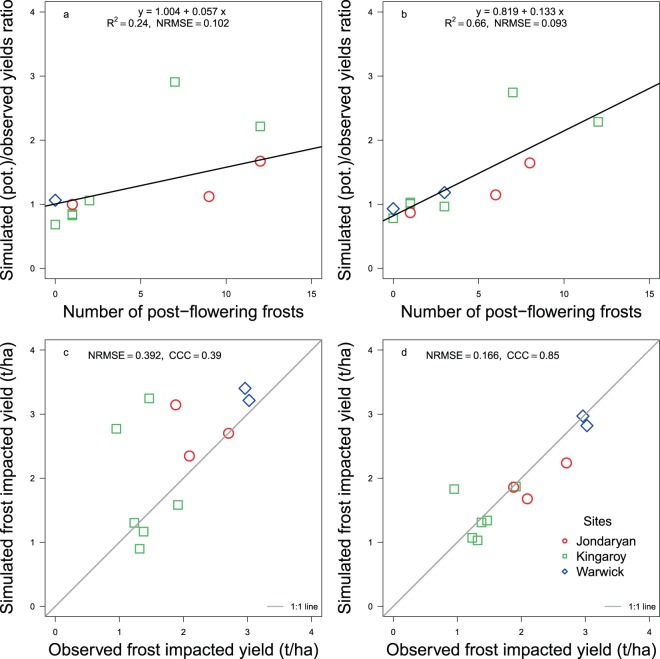
Relationship of (**a**) number of post-flowering frost events and the ratio of observed vs. simulated yield using original model, (**b**) improved model, (**c**) simulated frost impacted yield (5% loss/frost event) vs. observed yield using the original model and (**d**) with improved model. NRMSE is normalised root mean square error. The black line in (**a**,**b**) is the fitted regression and light grey line in c and d is y = x. CCC in (**c**,**d**) are Lin’s concordance correlation coefficient.

The improvement in accuracy with our modelling approach accounting for the soil water effect has agronomic implications as illustrated in the assessment of the risk of post-flowering frost (minimum temperature ≤0 °C) events^32^. The number of post-flowering frost events accounted for 24% of the variation in yield in the 11 sowings using the original model, whereas the model accounting for soil water increased it to 66% (Fig. 4a,b). The yield ratio in Fig. 4 represents the ‘gap’ between the potential and observed yields occurring due to frost. Decreasing the potential yield by 5% for each post-flowering frost event and relating the reduced yield to the observed yield, increased the Lin’s CCC from 0.39 with the original model to 0.85 with the model accounting for the soil water effect (Fig. 4c vs. 4d). The soil water based model to predict flowering time should therefore improve strategies to manage frost risk that could be expanded to other stresses.

### Prediction of flowering time of chickpea for more diverse ranges of environments

**Figure 5:**
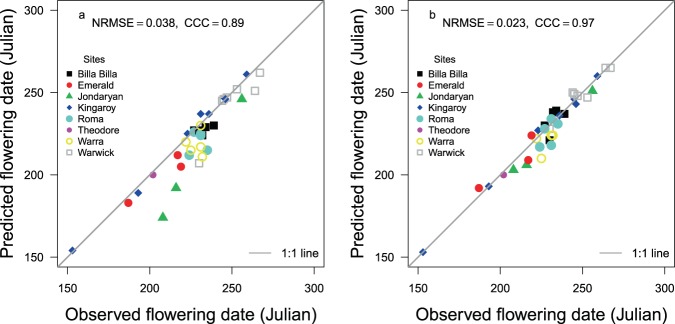
The predictive accuracy of flowering time for 37 chickpea sowings (**a**) based only on temperature and photoperiod and (**b**) based on the additional effect of soil water. NRSME is normalised root mean square error. The grey line is y = x. CCC = Lin’s concordance correlation coefficient.

We further compared the two modelling approaches, with and without soil water, using pooled data  including the original 11 sowings and 26 additional sowings with Pulse Breeding Australia (PBA) HatTrick from yield evaluation trials on soils of different plant available water holding capacities over a range of latitudes and longitudes within Queensland, Australia (Fig. 5). In these sowings, the improvement in Lin’s CCC was from poor (0.89) to substantial agreement categories (0.97).

We modelled chickpea flowering using a photoperiod range from 10 to 12 hours compared to the original range of 10 to 17 hours in APSIM^10^. Within chickpea growing regions and sowing windows adopted in Australia, photoperiod changes are within 2.5 hours before the September equinox by which time most chickpea have flowered. Hence, the rationale of using a higher range of photoperiod for Australian cultivars in the model may need re-examination. It might be that a larger photoperiod range was acting as a surrogate for the soil water effect. However, this would fail to capture locational and seasonal variation in soil water with variable influence on flowering time as shown in Figs 2 and 3. It is possible that photoperiod and temperature interactions, that are commonly reported in the field^8^ in chickpea, are actually three-way interactions between soil water, temperature and photoperiod as photoperiod by temperature interactions have not been observed in controlled environments^9,33^. Temperature interacts with soil water by influencing evapotranspiration demand. With the most divergences of flowering time in the model based on photoperiod and temperature falling below the 1:1 line (Figs 1a and 5a), it appears that temperature and photoperiod set the minimum time that a chickpea crop could take to flower. In contrast, soil water modulates the time between minimum and actual time to flower (Figs 1b and 5b). Soil water, however, is very dynamic, hence the importance of initial conditions in the modelling of flowering time incorporating soil water. We achieved reasonable improvement of prediction when we initialised soil water based on root zone water modelled by the Australian Landscape Water Balance on 1^st^ Nov, about six months before sowing. As an alternative, remote sensing can also be tested for soil water initialisation^34^.

### Prediction of flowering time of wheat

**Figure 6:**
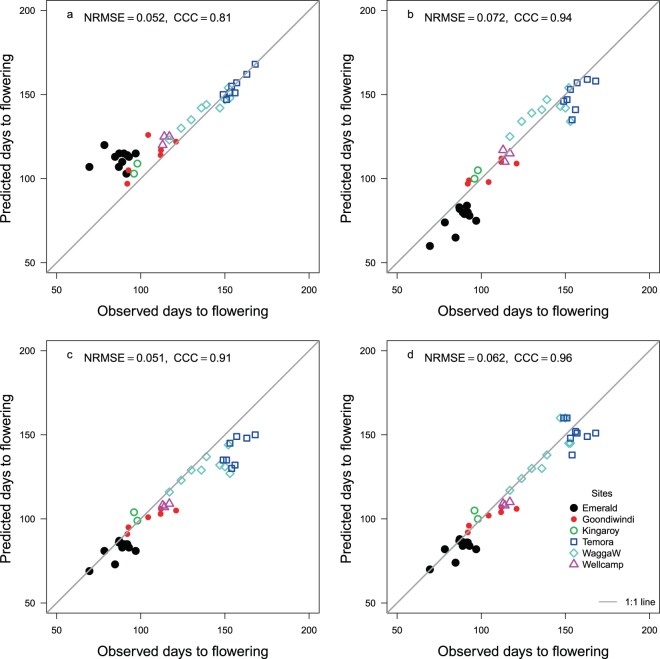
Observed and predicted days to flowering of the wheat cultivar Gregory using (**a**) the APSIM model with original parameters of photoperiod sensitivity and vernalisation, (**b**) Zheng, *et al*.^37^ model, (**c**) original model with reduced photoperiod and vernalisation sensitivity, (**d**) original model with reduced photoperiod and vernalisation sensitivity and the soil water effect. The Temora and WaagaW (Wagga Wagga) locations are in New South Wales and all others in Queensland. NRMSE is normalised root mean square error. The grey line is y = x. CCC = Lin’s concordance correlation coefficient.

Wheat is the dominant crop of southern Australian farming systems spread over southern New South Wales, Victoria and South Australia^35^. The application of the soil water correction improved the prediction of flowering time in wheat (Fig. 6). In APSIM, wheat flowering time is predicted based on temperature, photoperiod and vernalisation, and parameters accounting for these effects seem to have been optimised in those environments. For example, model parameters of the popular cultivar Enterprise Grains Australia (EGA) Gregory, which is less photo-period sensitive^36^, are such that the original APSIM predicts flowering reasonably well for New South Wales sites/sowings, but not so well for Queensland sites/sowings resulting in a poor (0.81) Lin’s CCC (Fig. 6a). The default value of photoperiod sensitivity factor for this cultivar in the model is set to 3.2 and vernalisation sensitivity to 2.7. Zheng, *et al*.^37^ reduced the photoperiod sensitivity factor of this cultivar to 2.6 and the vernalisation sensitivity to 0.9 using a gene-based parameterisation (Fig. 6b). This moderately improved the Lin’s CCC (0.94) although prediction of flowering time for the central Queensland location of Emerald required further improvement. The discrepancy of Emerald (central Queensland) sowings was reduced by increasing the vernalisation sensitivity from 0.90 to 1.98, but this change affected prediction of flowering time in New South Wales sowings (Fig. 6c). The Lin’s CCC increased to 0.96 when the soil water effect through Eq. 1 was incorporated (Fig. 6d). We can speculate that in the original model, the photoperiod and vernalisation parameters for this cultivar may have been fitted to overcome the soil water effect on flowering time for the dominant growing environments. Site-specificity of APSIM parameters for predicting wheat flowering which was suspected to occur due to the lack of proper model mechanisms has been highlighted earlier^38^. For a more robust prediction of flowering time, accounting for the soil water effect using the model we have developed may be necessary in wheat as well.

The use of the high photoperiod sensitivity parameter in the model for the otherwise relatively photoperiod insensitive wheat cultivar Gregory, as indicated above, may have unknowingly accounted for the soil water effect on flowering time at higher latitudes. In reality, however, soil water may be helping Gregory and other insensitive wheat cultivars to overcome some of the effects of earliness associated with photoperiod insensitivity. Photoperiod insensitivity in commercial wheat cultivars was introduced through the shuttle breeding of wheat at CIMMYT in Mexico in the 1960s^39^. Although Normal Borlaug considered photoperiod insensitivity an unplanned effect of shuttle breeding of wheat^40^, it is now recognised to have contributed to the green revolution in wheat as it enhanced adaptation of the crop to a wide range of latitudes^39^. The increase in pre-anthesis period of high yielding photoperiod insensitive wheat cultivars, achieved by the dynamic effect of soil water with irrigation, might have also assisted in avoiding frosts, as in the case of chickpea described above, and in producing more biomass while not having any of the adverse effects of photoperiod related sensitivity in yield components^41^.

### Concluding remarks

Our modelling study is consistent with, but does not prove, that soil water modulates flowering time. The effect of soil water on development is biologically interesting, and relevant for modelling and agronomy.

Biologically, the causes and consequences of this effect need further research. From an adaptive viewpoint, photoperiod is a conserved environmental cue; temperature follows seasonal patterns with some intra- and inter-seasonal variation, whereas soil water is characterised by larger temporal variability. We can thus hypothesise that photoperiod and temperature provide broad geographic adaptation, whereas soil water would fine-tune flowering time to specific soil-season combinations. At the plant level, part of the effect could be related to temperature, as a dry soil is hotter; this is particularly important for wheat as its growing apex is underground during key developmental stages^42^. Plants sense soil dryness and root-shoot signals are involved in modulation of shoot traits. Soil temperature has been suggested to affect flowering time in wheat^43^. However the possibility that soil temperature could affect flowering time in wheat was ruled out earlier through experiments involving manipulation of soil temperature^42^.

From a modelling perspective, we have shown that a model accounting for soil water, photoperiod and temperature is superior to conventional models in predicting flowering time. We have also shown the improvement in modelling risk associated with frost. Trade-offs between frost and heat damage are particularly important^35^, and could be modelled more reliably with our approach. Agronomically, practices can be envisaged to exploit genotype x environment x management interactions influencing soil water, and hence flowering time in a context of risk.

## Methods

### Soil water effect on flowering time in chickpea

We collected flowering and yield data from 11 chickpea sowings from 2014 to 2017 at Kingaroy (26.55°S and 151.85°E), Jondaryan (27.37 to 27.50°S and 151.59–151.76°E) and (Warwick 28.21°S and 152.10°E) in Queensland, Australia. These sowings were part of two trials conducted to compare response of chickpea cultivars PBA HatTrick (released in 2009) and PBA Boundary (released in 2011) to frosts and planting time. For sowing, a summer fallow was practiced for growing winter crops as in-season rainfall is often limiting. At the beginning of summer season (on 1^st^ Nov), it was assumed to contain very limited amount of soil water left by the previous crop. Since this initial amount was not measured, we used values obtained from the Australian Landscape Water Balance model (www.bom.gov.au/water/landscape). The soil water increased with subsequent summer rains. Sowings were at a 5 cm depth with adequate seed to achieve a plant population of 30 plants/m^2^. Other agronomic details are given in Table 1. Observations on flowering were made when 50% of the plants had at least one open flower. Yield at maturity was estimated from hand-harvested samples from a 2 m^2^ area.

### Prediction of flowering times using APSIM

The APSIM model (version 7.10)^44^ consists of many modules of different models that are called upon as needed, to predict flowering time. The model enables simulation of systems that cover a range of plant, soil, climate and management interactions. The plant module of APSIM provides uptake values for soil water to the soil module. The model uses a thermal time approach to predict flowering of chickpea^10^ using cultivar specific parameters that are included in the plant models^10^. In this study we used three chickpea cultivars including PBA Boundary, HatTrick, and Tyson (released in 1978) and their parameters including thermal time requirements for different phases of growth are given in Table 2. In the original model, the thermal time requirement for flowering of PBA Boundary and PBA HatTrick decreased from 446 to 0 °C during the photoperiod sensitive phase, as photoperiod increases from 10.7 to 17 h. Since chickpea does not experience more than 12 hours photoperiod from sowing to flowering in the winter season over a range of latitudes, we postulated that extra hours might have been built into the model to account for the soil water effect. Since we directly incorporated soil water information, we reduced the upper bound of photoperiod from 17 to 12 hours as given in Table 2. The same set of cultivar parameters was applied to all sowings. To incorporate the effect of soil water on flowering time we used the following equation in the manager module of the model:

1 $${{\rm{TT}}}_{{\rm{m}}}={\rm{TT}}\ast (1.65-{\rm{FASW}})\,({\rm{when}}\,{\rm{FASW}}\ge 0.65\,{\rm{and}}\,{\rm{the}}\,{\rm{chickpea}}\,{\rm{stage}}\ge 3)$$

Here TT_m_ is modified daily thermal time, TT is daily thermal time, and FASW is the fraction of available soil water in top 60 cm layers relative to lower limit at 1.5 MPa. These top layers constituted the effective rooting zone for chickpea where most of the roots are located^27,45^. TT in the model was computed using a set of cardinal temperatures with base = 0 °C, optimum = 30 °C and ceiling = 40 °C. TT equalled mean ambient temperature up to 30 °C. The ‘1.65’ in Eq. 1 was a constant identified through manual optimisation which limited reduction in TT only when FASW was >0.65. The available soil water in this range is described as the readily available water for chickpea and other crops^27^.

**Table 2:** Parameters of desi chickpea cultivars PBA HatTrick, and PBA Boundary and Tyson in the APSIM model.

Parameter	Range		Unit	Description
x_pp_hi_incr	1	24	h	Photoperiod
y_hi_incr	0.014	0.014	1/d	Rate of HI increase
x_hi_max_pot_stress	0	1		Average stress at flowering
y_hi_max_pot	0.5	0.5		Maximum harvest index potential
cum_vernal_days	0	100	d	
tt_emerg_to_endjuv	515^a^	515^a^	°Cd	TT from emergence to end of juvenile phase
est_days_emerg_to_init	83	d		Estimated days from emergence to floral initiation
x_pp_endjuv_to_init	10.7^b^	17^c^	h	Photoperiod
y_tt_endjuv_to_init	446^d^	0	°Cd	TT from end juvenile to floral initiation
x_pp_init_to_flower	1	24	h	Photoperiod
Y_tt_init_to_flower	33	33	°Cd	TT from initiation to flowering
x_pp_flower_to_start_grain	1	24	h	Photoperiod
y_tt_flower_to_start_grain	450	450	°Cd	TT from flowering to start grain fill
x_pp_start_to_end_grain	1	24	h	Photoperiod
y_tt_start_to_end_grain	690	690	°Cd	TT from start grain fill to end grain fill
tt_end_grain_to_maturity	60		°Cd	TT from end grain fill to maturity
tt_maturity_to_ripe	1		°Cd	TT from maturity to harvest ripe
x_stem_wt	0	10	g/plant	Stem weight
y_height	0	800	mm	Plant height

^a^Changed to 660 °Cd for PBA Boundary and PBA HatTrick, 690 °Cd for Tyson. ^b^10.1 h for Tyson. ^c^Changed to 12 h in the new model, ^d^468.3 °Cd for Tyson.

FASW was computed in the manager module of APSIM as a ratio of extractable water and the total available water. The soil module, which is a cascading water balance model, simulates the relevant processes in the soil profile including soil water infiltration, movement, evaporation, runoff, drainage, extractable soil water and the total available water. To determine water balance, the model uses an input of 1.5 MPa lower limit (ll15 – the driest water achievable by plant water extraction), and drained upper limit (DUL, field capacity), saturated volumetric water, stage 1 and 2 evaporation parameters, and water uptake, all were computed by the plant module in a daily time step. These and other related parameters, except for water uptake by plants, for soils which were used in this study, were obtained through systematic soil sampling and characterisation. These are available in the APSoil database. The soil water module was called in APSIM on a daily basis to compute FASW using the following equation:

2 $${\rm{FASW}}={\rm{\Sigma }}({\rm{sw}}\_{\rm{dep}}({\rm{i}})-{\rm{ll}}15\_{\rm{dep}}({\rm{i}}))\_/{\rm{\Sigma }}({\rm{dul}}\_{\rm{dep}}({\rm{i}})-{\rm{ll}}15\_{\rm{dep}}({\rm{i}}))$$

where sw_dep is soil water, ll15_dep is the soil water corresponding to a soil water potential of 1.5 MPa, and dul_dep is the soil water at field capacity (0.03 MPa) in each layer (i) in the top 60 cm soil surface layers. Equation 1 was operationalised only when FASW was >0.65 and the emergence (growth stage 3) had occured. The reduction in TT was maximum when FASW values were ≥1, i.e., when soil water in the surface 60 cm layers was near the field capacity.

The extractable soil water was defined as the water held between field capacity (DUL) and at 1.5 MPa soil water potential (ll15) in the soil. Soil parameters of all sowing locations except for Jondaryan in 2015 were obtained from the APSoil database (www.apsim.info). For the Jondaryan 2015 sowing, a generic Vertisol (No. 523) of 137 mm water available water holding capacity was selected from the same database based on water holding capacity described for an agricultural area around a nearby mining site^46^. Daily data from nearby weather stations were downloaded from the apsrunet.apsim.info website. Weather at the experimental sites was also monitored in the 11 sowings, which was patched on to the weather data downloaded from the apsurnet.apsim.info website. The user interface of APSIM was configured to link the above soil and daily weather data and other agronomic details including cultivar, sowing date, depth of sowing, row spacing and plant population used. The change in accumulated thermal time was accomplished on a daily basis through the manager module of the model. Flowering time was also simulated using unmodified model parameters.

### Assessment of the effect of post flowering frosts on chickpea yield

In the above-mentioned 11 chickpea sowings, the crop experienced a varying number of frost events (≤0 °C) before and after flowering in all three locations. The number of post-flowering frosts computed by the model was contingent upon the accuracy of prediction of flowering time. We assessed the impact of frost on yield using two approaches. In the first approach, we compared the relationship between the number of frost events and the ratio of potential realisable yield and the observed yield, which represented the yield gap. In the second approach, we compared the observed yield with the simulated yield loss due to post-flowering frosts. It has been estimated that each post-flowering frost event causes about 5% loss in yield of chickpea. The following equations were, therefore, incorporated in the manager module to simulate 5% yield loss for each post-flowering frost event:

3 $${{\rm{Yield}}}_{{\rm{L}}}={{\rm{Yield}}}_{{\rm{W}}}\ast ({\rm{\Sigma }}\mathrm{PFF}\ast 0.05)$$

4 $${{\rm{Yield}}}_{{\rm{GM}}}={{\rm{Yield}}}_{{\rm{w}}}-{{\rm{Yield}}}_{{\rm{L}}}$$

Yield_L_ is the yield lost due to frost, Yield_W_ is yield with 12% seed moisture content (potential yield), and Yield_GM_ is the observed (gross margin) yield a grower would harvest. PFF is a post-flowering frost event when the minimum temperature was ≥0 °C.

### Validation of the soil water model to predict flowering time in chickpea in diverse range of sowings and seasons

Flowering was also predicted in a larger set of 24 additional sowings from breeding yield trials and two farming systems trials (Peter Want, DAF, Kingaroy, Personal Communication) conducted from 2013 to 2017 in Queensland. These locations covered 23.4 to 28.5°S latitude and 148.1 to 152.1° longitude. The set up procedure of the model was similar to that for the 11 sowings of chickpea. The soil parameters for the simulations were obtained from the APSoil database and weather data from the apsurnet.apsim.info website. The details of these sowings and soils are given in the supplementary information (Supplementary Table 1). Simulations of all sowings were initialised on 1^st^ Nov using data obtained from the Australian Landscape Water Balance (Supplementary Table 1).

Simulations were similarly run to predict flowering times in rainfed trials conducted in 1979 and 1980^29^ at Dalby, Queensland. Soil parameters for the site were obtained from the APSoil data base and weather data from the apsurnet.apsim.info website (Supplementary Table 1). Simulations were initialised on 1^st^ Nov of the previous year with a low soil water of 20% as landscape water balance data were not available for those two seasons. Other agronomic information included in the model was as described in the papers^29,30^. Weather data were obtained from the apsurnet.apsim.info website.

### Soil water effect on flowering time in wheat

The applicability of Eq. 1 to predict flowering of wheat was tested for cultivar Gregory grown at Goondiwindi, Emerald, Kingaroy, and Wellcamp in Queensland, and Wagga Wagga and Temora in New South Wales spread over 23.5 to 35.0°S latitude and 147.3 to 151.9° longitude. FASW was computed using the APSIM model as described for chickpea in Eq. 1 to predict flowering time. The soil parameters for the simulations were obtained from the APSoil database (Supplementary Table 2). Soil water was initialised to a low level on 1^st^ Nov of the previous year using the Australian Soil Water balance model (www.bom.gov.au/water/landscape). The model set up was similar to that for chickpea. All observed flowering data were either obtained from the Grains Research and Development Corporation website www.grdc.com.au and for Kingaroy from a colleague (Peter Want, DAF, Kingaroy, Personal Communication). The APSIM-Wheat is a process-oriented modular structure with externalised model parameters^47^. To predict flowering time, the daily thermal time in the model was reduced by multiplying it with the vernalisation and photoperiod sensitivity factors for a given cultivar (www.apsim.info). Cultivar Gregory released in 2004 for cultivation in Queensland and New South Wales was considered to be relatively photoperiod insensitive^36^, but it has high a photoperiod factor of 3.2 (for 0 to 5 scale) in the model. We hypothesized that if Gregory cultivar is indeed photoperiod insensitive, the high photoperiod sensitivity for this cultivar in the APSIM model could be to account for the effect of soil water on flowering. Zheng, *et al*.^37^ through actual field experiments estimated its photoperiod sensitivity factor as 2.6. They also reduced its vernalisation sensitivity from 2.7 to 0.9 while increasing its thermal time to floral initiation requirement from 555 to 715 °C days. We compared prediction of flowering of wheat in 40 sowings/site combinations using the original APSIM model, the APSIM model proposed by Zheng, *et al*.^37^ and the soil water based model as proposed in this study.

The details of these sowings are given in the supplementary information (Supplementary Table 2). While applying the soil water based model using Eq. 1, the vernalisation sensitivity of the original APSIM model was reduced from 2.7 to 1.98, and the photoperiod sensitivity to 2.6 as used for Gregory in Zheng, *et al*.^37^ while the thermal time to initiation was kept as 555 °C days. These changes were derived from manual optimisation.

### Statistical analysis

The relationship between observed/simulated yield and simulated yield after reducing it by 5% for each post-flowering frost event (as y variable), was quantified using linear regression with the R program^48^. The normalised root mean square error (NRMSE) in Figs 1, 4, 5 and 6 was computed using the following equation in the same program.

5 $$\begin{array}{c}{\rm{N}}{\rm{R}}{\rm{M}}{\rm{S}}{\rm{E}}=\surd ({\rm{m}}{\rm{e}}{\rm{a}}{\rm{n}}({\rm{f}}{\rm{i}}{\rm{t}}{\rm{t}}{\rm{e}}{\rm{d}}{\rm{\_}}{\rm{f}}{\rm{l}}{\rm{o}}{\rm{w}}{\rm{e}}{\rm{r}}{\rm{i}}{\rm{n}}{\rm{g}}-{\rm{s}}{\rm{i}}{\rm{m}}{\rm{u}}{\rm{l}}{\rm{a}}{\rm{t}}{\rm{e}}{\rm{d}}{\rm{\_}}{\rm{f}}{\rm{l}}{\rm{o}}{\rm{w}}{\rm{e}}{\rm{r}}{\rm{i}}{\rm{n}}{\rm{g}}{)}^{2})\\ \,\,\,\,\,\,\,/{\rm{m}}{\rm{e}}{\rm{a}}{\rm{n}}\,({\rm{o}}{\rm{b}}{\rm{s}}{\rm{e}}{\rm{r}}{\rm{v}}{\rm{e}}{\rm{d}}{\rm{\_}}{\rm{f}}{\rm{l}}{\rm{o}}{\rm{w}}{\rm{e}}{\rm{r}}{\rm{i}}{\rm{n}}{\rm{g}})\end{array}$$

Lin’s concordance correlation coefficient (CCC) ρ_c_^28,49^ reported in Figs 1, 4, 5 and 6 was used to quantify a model’s predictive performance. Lin’s CCC achieves this by measuring how well the relationship between the observations and model predictions is represented by a straight line through the origin at an angle of 45 degrees. Lin’s CCC is defined as

6 $${{\rm{\rho }}}_{{\rm{c}}}={\rm{\rho }}\times {{\rm{C}}}_{{\rm{b}}}$$

where ρ is the Pearson product-moment correlation coefficient and C_b_ is a bias correction factor calculated as

7 $${{\rm{C}}}_{{\rm{b}}}=2/({\rm{v}}+1/{\rm{v}}+{{\rm{u}}}^{2})$$

8 $${\rm{v}}={{\rm{s}}}_{1}/{{\rm{s}}}_{2}$$

9 $${\rm{u}}=({{\rm{m}}}_{1}-{{\rm{m}}}_{2})/\surd ({{\rm{s}}}_{1}\times {{\rm{s}}}_{2})$$

where m_i_ and s_i_ (i = 1, 2) are the mean and standard deviation of the observations (i = 1) and predictions (i = 2). McBride^50^ suggests the following guidelines to infer a model’s predictive performance.

- ρ_c_ < 0.90: poor
- ρ_c_ > 0.90 to 0.95: moderate
- ρ_c_ > 0.95 to 0.99: substantial
- ρ_c_ > 0.99 almost perfect.

## Supplementary information

Accounting for soil moisture improves prediction of flowering time in chickpea and wheat
