# Supplementary material for: Accounting for soil moisture improves prediction of flowering time in chickpea and wheat
Source: Sci Rep. 2019 May 17;9:7510. doi: 10.1038/s41598-019-43848-6 (PMC6525173; doi:10.1038/s41598-019-43848-6)
Supplement: Supplementary file 1 — Accounting for soil moisture improves prediction of flowering time in chickpea and wheat [file 41598_2019_43848_MOESM1_ESM.docx]

Accounting for soil moisture improves prediction of flowering time in chickpea and wheat

Yashvir S. Chauhan^1*^, Merrill Ryan^2^, Subhash Chandra^3^ & Victor O. Sadras^4^

*^1^Department of Agriculture and Fisheries (DAF), Kingaroy, Queensland 4610, Australia*

*^2^DAF, Hermitage Research Station, 604 Yangan Road, Warwick, Queensland 4370*

*^3^Agriculture Research Division, Agriculture Victoria, 255 Ferguson Road, Tatura 3616, Victoria, Australia*

*^4^South Australian Research and Development Institute, Adelaide, Australia*

**Supplementary Table 1**: Details of site, latitude, longitude, sowing date, cultivar, initial water, soil, and plant available water-holding capacity (PAWC) of the soil for chickpea^a^.

Lat. Long. Initial water Soil PAWC

Site °Dec . °Dec. Sowing date Cultivar (%, 1^st^ Nov) (APSoil no)^b^ (mm)

Billa Billa -28.2 150.4 4-Jun-13 Hattrick 7 Grey Vertosol (229) 183

Billa Billa -28.2 150.4 26-May-14 Hattrick 7 Grey Vertosol (229) 183

Billa Billa -28.2 150.4 27-May-15 Hattrick 7 Grey Vertosol (229) 183

Billa Billa -28.2 150.4 24-May-16 Hattrick 23 Grey Vertosol (229) 183

Billa Billa -28.2 150.4 29-May-17 Hattrick 23 Grey Vertosol (229) 183

Dalby^b^ -27.2 151.3 17-May-79 Tyson 20 Black Vertosol-Bongeen (27) 199

Dalby^b^ -27.2 151.3 1-Jun-80 Tyson 20 Black Vertosol-Bongeen (27) 199

Emerald^c^ -23.5 148.1 6-May-15 Hattrick 12 Shallow grey Vertosol (1266) 153

Emerald^d^ -23.5 148.1 25-May-16 Hattrick 12 Shallow grey Vertosol (1266) 153

Emerald^e^ -23.5 148.1 2-Jun-17 Hattrick 12 Shallow grey Vertosol (1266) 153

Kingaroy -26.6 151.8 9-Jun-16 Hattrick 32 Red Ferrosol (107) 109

Kingaroy -26.6 151.8 20-Jun-17 Hattrick 23 Red Ferrosol (107) 109

Roma -26.5 148.8 28-May-13 Hattrick 7 Brown Vertosol (63) 119

Roma -26.5 148.8 15-May-14 Hattrick 7 Brown Vertosol (63) 119

Roma -26.5 148.8 26-May-15 Hattrick 7 Brown Vertosol (63) 183

Roma -26.5 148.8 19-May-16 Hattrick 17 Brown Vertosol (63) 119

Roma -26.5 148.8 30-May-17 Hattrick 17 Brown Vertosol (63) 119

Theodore -24.9 150.1 17-May-17 Hattrick 7 Black Vertosol(112) 129

Warra -26.9 150.9 29-May-13 Hattrick 12 Grey Vertosol (115) 207

Warra -26.9 150.9 16-May-14 Hattrick 12 Grey Vertosol (115) 207

Warra -26.9 150.9 25-May-15 Hattrick 12 Grey Vertosol (115) 207

Warra -26.9 150.9 18-May-16 Hattrick 17 Grey Vertosol (115) 207

Warra -26.9 150.9 24-May-17 Hattrick 12 Grey Vertosol (115) 207

Warwick -28.2 152.1 10-Jun-14 Hattrick 23 Brown Vertosol (33) 216

Warwick -28.2 152.1 26-Apr-15 Boundary 7 Brown Vertosol (33) 216

Warwick -28.2 152.1 3-Jun-15 Hattrick 7 Brown Vertosol (33) 216

Warwick -28.2 152.1 14-Jun-16 Hattrick 17 Brown Vertosol (33) 216

Warwick -28.2 152.1 9-Jun-17 Hattrick 32 Brown Vertosol (33) 216

^a^ All sowings were done at 30 plants/m^2^, APSoil – <http://apsrunet.apsim.info/>; ^b^ Dalby site only used for drawing figure 2, and other sites used for Fig. 3; ^c^ 100 mm irrigation given on 21-Apr-15; ^d^ 100 mm irrigation given on 9-May-16; and ^e^ 75 mm irrigation given on 23-Apr-17, all other sites were rainfed.

**Supplementary Table 2**: Details of site, latitude, longitude, sowing date, cultivar, initial water, soil, and plant available water- holding capacity (PAWC) of the soil for wheat^a^

Site Latitude Longitude Sowing Initial water Soil PAWC

°Dec. °Dec. date (%, 1^st^ Nov) (APSOIL no.)^b^ (mm)

Emerald -23.5 148.1 1-May-15 12 Black Vertosol (911) 287

Emerald -23.5 148.1 7-May-15 12 Black Vertosol (911) 287

Emerald -23.5 148.1 8-May-15 12 Black Vertosol (911) 287

Emerald -23.5 148.1 22-Apr-16 12 Black Vertosol (911) 287

Emerald -23.5 148.1 29-Apr-16 12 Black Vertosol (911) 287

Emerald -23.5 148.1 29-Apr-16 12 Black Vertosol (911) 287

Emerald -23.5 148.1 6-May-16 12 Black Vertosol (911) 287

Emerald -23.5 148.1 6-May-16 12 Black Vertosol (911) 287

Emerald -23.5 148.1 2-May-17 12 Black Vertosol (911) 287

Emerald -23.5 148.1 3-May-17 12 Black Vertosol (911) 287

Emerald -23.5 148.1 3-May-17 12 Black Vertosol (911) 287

Emerald -23.5 148.1 4-May-17 12 Black Vertosol (911) 287

Goondiwindi -28.5 150.3 2-May-15 12 Grey Vertosol (219) 162

Goondiwindi -28.5 150.3 24-May-15 12 Grey Vertosol (219) 162

Goondiwindi -28.5 150.3 4-Jun-15 12 Grey Vertosol (219) 162

Goondiwindi -28.5 150.3 11-May-16 23 Grey Vertosol (219) 162

Goondiwindi -28.5 150.3 27-May-16 23 Grey Vertosol (219) 162

Goondiwindi -28.5 150.3 2-Jun-16 23 Grey Vertosol (219) 162

Kingaroy -26.6 151.8 22-May-16 32 Red Ferrosol (107) 109

Kingaroy -26.6 151.8 11-May-17 23 Red Ferrosol (107) 109

Temora -34.4 147.5 22-Jun-15 7 Red Sodosol (823) 179

Temora -34.4 147.5 27-Jun-15 7 Red Sodosol (823) 179

Temora -34.4 147.5 3-Jul-15 7 Red Sodosol (823) 179

Temora -34.4 147.5 10-Jul-15 7 Red Sodosol (823) 179

Temora -34.4 147.5 28-Jun-16 12 Red Sodosol (823) 179

Temora -34.4 147.5 2-Jul-16 12 Red Sodosol (823) 179

Temora -34.4 147.5 6-Jul-16 12 Red Sodosol (823) 179

Temora -34.4 147.5 12-Jul-16 12 Red Sodosol (823) 179

Wagga Wagga -35.0 147.3 9-Jun-12 7 Red Brown Earth (538-YP) 66

Wagga Wagga -35.0 147.3 17-Jun-12 7 Red Brown Earth (538-YP) 66

Wagga Wagga -35.0 147.3 30-Jun-12 7 Red Brown Earth (538-YP) 66

Wagga Wagga -35.0 147.3 3-Jun-13 7 Red Brown Earth (538-YP) 66

Wagga Wagga -35.0 147.3 8-Jun-13 7 Red Brown Earth (538-YP) 66

Wagga Wagga -35.0 147.3 21-Jun-13 7 Red Brown Earth (538-YP) 66

Wagga Wagga -35.0 147.3 23-Jun-16 17 Red Brown Earth (538-YP) 66

Wagga Wagga -35.0 147.3 30-Jun-16 17 Red Brown Earth (538-YP) 66

Wagga Wagga -35.0 147.3 3-Jul-16 17 Red Brown Earth (538-YP) 66

Wellcamp -27.5 151.9 27-May-17 23 Black Vertosol (30) 244

Wellcamp -27.5 151.9 3-Jun-17 23 Black Vertosol (30) 244

Wellcamp -27.5 151.9 8-Jun-17 23 Black Vertosol (30) 244

^a^Wheat cultivar Gregory was sown at 100 plants/m^2^ on 250 mm rows. ^b^APSoil – <http://apsrunet.apsim.info/>
